# Diagnostic Yield of Continuous Video EEG for Neonatal Seizures

**DOI:** 10.15844/pedneurbriefs-29-8-6

**Published:** 2015-08

**Authors:** Tammy N. Tsuchida

**Affiliations:** 1 Division of Neurophysiology, Epilepsy and Critical Care in Center for Neuroscience and Behavioral Health, Children's National Health System, Washington, DC; 2 Departments of Neurology and Pediatrics, George Washington University School of Medicine and Health Sciences, Washington, DC

**Keywords:** Critical care, Neonatal seizures, EEG, Neonatal encephalopathy

## Abstract

Investigators from the University of California, San Francisco studied the yield of continuous video EEG (vEEG) in diagnosing electrographic seizures in their neonatal intensive care unit.

Investigators from the University of California, San Francisco studied the yield of continuous video EEG (vEEG) in diagnosing electrographic seizures in their neonatal intensive care unit. Over a 4.5 year period, 595 neonates were evaluated, of which 66% were term and 67% referred from an outside hospital. Therapeutic hypothermia was completed in 25%. There was a 14% mortality rate. Neonates with electrographic seizures were identified by reviewing clinical vEEG reports.

vEEG was clinically indicated for 400/595 (67%) of the neonates, with approximately equal proportions for two or more of the following indications: event concerning for seizure, encephalopathy, or high risk for seizures. Continuous vEEG was performed for a median of 49 hours (interquartile range 22-87). All neonates undergoing therapeutic hypothermia received vEEG until rewarmed. Electrographic seizures were detected in 105/400 (26%), and of those 25/105 (24%) had only electrographic seizures, with no clinical seizures even prior to vEEG. No seizures were detected on vEEG in 52/400 (13%) of those with events concerning for seizure. Phenobarbital was given prior to vEEG in 38/51 (75%) of those patients and to 93/400 (23%) of the entire study population.

The indication for vEEG did not affect the likelihood of seizure diagnosis. There was some variability in seizure diagnosis based on etiology. Arterial and venous strokes had the highest proportion with seizures in 58%, and hypoxic-ischemic encephalopathy, intracranial hemorrhage and infection all around 29% and lower rates with brain malformation or genetic syndromes. [1]

COMMENTARY. Continuous vEEG is increasingly utilized in the intensive care setting. As discussed by the authors, there is variability in the use of EEG with 40-70% of neonatologists and neurologists using EEG or amplitude integrated EEG (aEEG) to evaluate at risk infants [1]. There is literature to support the use of continuous vEEG for at risk neonates. Of those neonates at high risk for seizures, 45-51% had seizures, of which 26-41% were only electrographic [2, 3]. After major cardiac surgery, 11-20% have seizures, and 59-100% have electrographic only seizures [4, 5]. Of neonates undergoing hypothermia for hypoxia-ischemia 34-65% have seizures and 43-47% are only electrographic [1]. As discussed by the authors, there are also studies that show video EEG is needed to correctly identify neonates with electroclinical seizures1. These studies have been of moderate size, with 51-183 neonates.

The authors discuss limitations of the study including inability to confirm how many clinical seizures might have had electrographic correlate prior to treatment with phenobarbital and potential over-representation of infants at higher risk for seizures. In addition, the systematic use of continuous vEEG for all neonates undergoing hypothermia could introduce screening bias.

This large study is consistent with earlier studies demonstrating a large proportion of neonates with electrographic seizures, many of whom would only be detected by vEEG. In addition, there are neonates with suspected seizures that are not confirmed on vEEG. This supports the use of continuous vEEG to optimize treatment of neonatal seizures. With continuous vEEG, one can ensure that treatment is only given to those with EEG confirmed seizures. And those with seizures detectable only with EEG will be identified and receive seizure treatment.
